# Possible Donor-Derived Infection in a Pediatric Liver Transplant Patient With Granulomatous Hepatitis

**DOI:** 10.7759/cureus.49136

**Published:** 2023-11-20

**Authors:** Lea R Goren, Oyedele Adeyi, Beth K Thielen

**Affiliations:** 1 Pediatric Infectious Diseases, University of Minnesota School of Medicine, Minneapolis, USA; 2 Laboratory Medicine and Pathology, University of Minnesota School of Medicine, Minneapolis, USA

**Keywords:** metagenomic next-generation sequencing, liver transplant, immunocompromised, pediatric, adenovirus hepatitis

## Abstract

Pediatric liver transplant recipients are a high-risk group for the development of adenovirus hepatitis and other manifestations of disseminated adenoviral disease. The risk is greatest during periods of increased immunosuppression, including immediately post-transplantation and following treatment for rejection. Manifestations of adenovirus hepatitis are heterogeneous with a wide spectrum of clinical severity, ranging from mild, focal disease to fulminant liver failure. Here we report a case of liver transplantation-associated adenovirus hepatitis presenting with fever and multifocal liver lesions. The diagnosis was not clinically suspected due to atypical imaging findings and pathology. Non-targeted metagenomic sequencing of plasma cell-free DNA facilitated and expedited the diagnosis. Confirmatory conventional testing was obtained, allowing for appropriate initiation of targeted treatment in this patient.

## Introduction

Every year in the United States, 500-600 children undergo liver transplantation for the treatment of conditions including biliary atresia, inborn errors of metabolism, acute liver failure, and hepatoblastoma [1]. Recipients of liver and other solid organ transplants are at increased risk for severe infections due to immune-suppressing medications they receive to prevent rejection of the transplanted organ(s). Prolonged and severe viral infections are of particular risk because similar pathways of cell-mediated immunity are involved in both graft rejection and control of viral infections. Thus, inhibition of these pathways for therapeutic benefit has the side effect of increasing susceptibility to viral infections. Among these viral pathogens are the adenoviruses, which are linear, double-stranded DNA viruses with seven described species (denoted A-G) and over 60 serotypes [2]. Adenoviruses infect multiple mucosal sites and are readily transmissible via multiple routes, including inhalation of aerosolized respiratory droplets, direct contact with mucous membranes, and ingestion of stool-contaminated food or water, a feature likely responsible for their ability to cause outbreaks among people in crowded environments. Adenovirus infections most commonly present with fever and respiratory or gastrointestinal symptoms in immunocompetent hosts, but more severe manifestations with end-organ involvement, including hepatitis and disseminated disease, are known complications in solid organ transplant recipients [3]. Rarely, donor-derived sources of infection have been documented [4,5]. Here, we report an unusual case of a suspected donor-derived infection presenting with focal liver lesions with atypical multifocal granulomatous inflammation without characteristic adenovirus inclusions on histopathology.

## Case presentation

The patient was a 12-month-old, 7.9 kg male with a history of biopsy-confirmed biliary atresia status post failed hepatoportoenterostomy at two months of age and orthotopic liver transplantation two weeks earlier, who was admitted for diagnostic evaluation of new fevers.

One month prior to his current presentation, he had a separate febrile illness associated with human rhinovirus/enterovirus (RV/EV) detection on a nasal respiratory panel (ePlex® Respiratory Pathogen Panel, GenMark Dx, Carlsbad, California) and acute Epstein-Barr virus (EBV) infection, with negative EBV serologies and positive EBV whole blood DNA PCR (52,052 copies/mL). Although blood cultures remained sterile and liver enzymes remained at baseline, he completed an empiric course of therapy with piperacillin-tazobactam for possible cholangitis, and fevers completely resolved.

Two weeks prior to his current presentation, he underwent deceased-donor liver transplantation with a cytomegalovirus (CMV)-positive and EBV-positive organ. The immediate post-transplant course was uncomplicated except for leukocytosis attributed to steroids and transient bradycardia attributed to the lingering effects of dexmedetomidine. He was afebrile throughout hospitalization and discharged on postoperative day 11. He received induction immunosuppression with basiliximab, methylprednisolone, and mycophenolate mofetil and continued on maintenance immunosuppression with tacrolimus, mycophenolate mofetil, and prednisone at the time of discharge. Post-transplant infection prophylaxis included valganciclovir, trimethoprim-sulfamethoxazole, and nystatin.

On the day of the current admission, he became febrile and returned to the hospital. At this time, blood and urine cultures were collected, and piperacillin-tazobactam (75 mg/kg q6h) and vancomycin (15 mg/kg q6h) were empirically started. Ultrasound of the transplanted liver revealed normal echotexture and blood flow by Doppler. On hospital day 2, a nasal respiratory panel was again positive for RV/EV, newly positive for a non-SARS-CoV-2 coronavirus, and negative for adenovirus. EBV PCR remained elevated (8897 copies/mL) but was decreased from the most recent test pretransplant. CMV blood PCR was undetectable.

**Figure 1:**
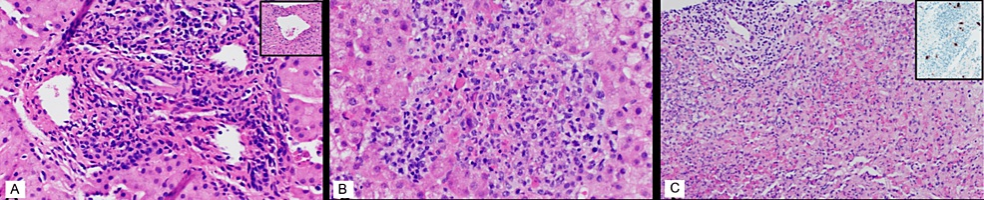
Histopathological images from three consecutive liver biopsies Representative hematoxylin and eosin-stained sections are included in each of the three main panels.  The first biopsy from day 6 (A) shows features of acute cellular rejection, characterized by portal mixed inflammation with portal phlebitis and hepatic vein phlebitis (A, inset). Repeat random biopsy on day 12 shows resolved rejection but small clusters of lymphohistiocytic inflammatory aggregates (B). Three days later (day 15), a targeted biopsy directed at mass-like lesions in the liver demonstrates larger lymphohistiocytic aggregates with necrosis and loose granulomas (C). Immunohistochemistry targeting adenovirus proteins shows increasing numbers of brown-staining cells over serial biopsies, progressing from isolated brown-staining cells on hospital day 6 to clusters of brown-staining cells on day 15 (C, inset). (Original magnifications A-C 200×; insets 100×).

Over the following days, he experienced resolution of fever but worsening of liver tests: ALT 132 U/L (reference range 0-50 U/L), AST 133 U/L (reference range 0-60 U/L), GGT 228 U/L (reference range 0-30 U/L), and direct bilirubin 0.6 mg/dL (reference range 0.0-0.2 mg/dL). This prompted an ultrasound-guided random core needle biopsy on hospital day six due to concern for acute rejection. The biopsy revealed moderate-to-severe acute cellular rejection (Figure 1A). At this point, high-dose IV methylprednisolone (20 mg/kg) was initiated, and empiric antimicrobials were stopped on hospital day 8.

**Figure 2:**
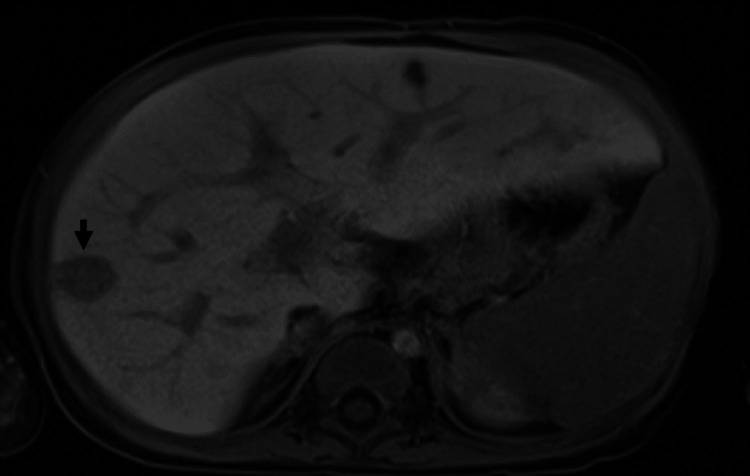
Representative section of a magnetic resonance imaging scan of the liver The largest hepatic lesion, measuring 1.6 x 1.4 cm, is indicated by the black arrow.

Due to recurrent fevers and rising C-reactive protein (30.0 mg/L, reference range 0.0-8.0 mg/L) and transaminases levels on hospital day 10, empiric antimicrobials were restarted (piperacillin-tazobactam 60 mg/kg q6h, vancomycin 15 mg/kg q6h, micafungin 3 mg/kg daily). A repeat liver ultrasound revealed two new hypoechoic lesions in the right lobe. Magnetic resonance imaging was performed for better visualization of the transplanted liver and revealed numerous T1 hypointense and T2 hyperintense lesions with mild diffusion restriction (Figure 2).

These lesions were suspicious for multifocal infection, prompting repeat ultrasound-guided core needle biopsy on hospital day 12. No organisms or evidence of ongoing rejection was seen (Figure 1B), so steroids were weaned. Because of ongoing fevers, a third core needle biopsy was performed on hospital day 15 under computed tomography guidance to accurately sample a lesion. Pathology showed larger lymphohistiocytic aggregates with necrosis and loose granulomas (Figure 1C), but cultures and stains for infectious causes including CMV, EBV, herpes simplex virus, and bacterial, fungal, and mycobacterial organisms were negative. No findings of post-transplant lymphoproliferative disorder were observed.

Due to nondiagnostic infectious disease testing, ongoing fevers, and rising C-reactive protein (maximum 83.7 mg/L, reference range 0.0-8.0 mg/L), metagenomic next-generation sequencing of microbial cell-free DNA from plasma (Karius Test®, Karius, Redwood City, CA) was sent on hospital day 16, and the pediatric infectious diseases service was consulted. Additional infectious workup returned negative, including Aspergillus galactomannan; 1,3-β-D-glucan; Histoplasma, Blastomyces, and Cryptococcus antigens; Toxoplasma PCR; and Treponema, Coxiella, Borrelia burgdorferi (Lyme disease), and Bartonella serologies. Metagenomic next-generation sequencing returned positive for a species C adenovirus (110,575 DNA molecules/µL) and Torque teno virus 1 (839 DNA molecules/µL) on hospital day 21. Torque teno virus is not typically thought to be pathogenic, so no further action was taken based on this result. Quantitative testing of blood (548,000 copies/mL whole blood) and repeat nasal respiratory secretions sent on hospital day 21 also returned positive for human adenovirus. Adenovirus stool antigen testing was negative. Retrospective immunohistochemical staining of all three liver biopsies revealed adenovirus-positive cells at increasing frequency over time, despite the absence of characteristic viral inclusions (Figure 1C, inset). Due to confirmed symptomatic adenovirus infection, mycophenolate was held and a lower permissible tacrolimus trough goal was set (7 vs. 10 µg/mL). A single dose of cidofovir (5 mg/kg) was administered with probenecid and hydration on hospital day 22. The patient became afebrile on hospital day 27 with an associated decrease in adenovirus whole blood quantitative PCR value to 42,540 copies/mL on hospital day 29 and 6,750 copies/mL on hospital day 32. Repeat ultrasound on hospital day 34 showed resolution of previously seen echogenic lesions. He required no additional antiviral therapy in light of steady clinical and virological improvement and was discharged on hospital day 34. Repeat adenovirus PCR from blood was undetectable when measured seven weeks after discharge.

## Discussion

Though adenoviruses are generally mild, self-limited infections in immunocompetent hosts, immunocompromised patients are at risk for more severe manifestations, including disseminated disease and hepatitis, which require more thorough diagnostic evaluation and, in some cases, antiviral treatment. The diagnosis of hepatitis in liver transplant recipients is typically established based on clinical features of fever, elevated transaminase levels, and characteristic histopathology of adenovirus inclusions on liver biopsy [6,7]. In a recent case series of acute hepatitis of unknown etiology in a pediatric cohort, the median adenovirus viral load was higher in patients with acute liver failure compared to those without acute liver failure, suggesting a positive association between adenovirus viral load and disease severity [8].

In this case, an adenovirus infection was not initially considered high in our differential due to multifocal granulomatous inflammation and the absence of characteristic adenovirus inclusions on pathology. However, adenovirus-specific immunohistochemistry, even in the absence of inclusions, exhibits increased sensitivity for end-organ disease, as previously described in a renal transplant recipient [9] and also illustrated by our case. In a recent case series of 44 previously healthy pediatric patients with acute hepatitis of unknown cause, human adenovirus was isolated in most children (27 out of 30 tested) [10]. Interestingly, out of the nine liver samples obtained in this cohort, no viral inclusions were noted, similar to our patient. The most commonly described pathological finding of adenovirus hepatitis is coagulative hepatocyte necrosis, ranging from focal to massive and often without inflammation. However, focal lymphohistiocytic inflammation has been noted, including in a 52-year-old man with chronic lymphocytic leukemia, who presented with multiple necrotizing granulomas and adenovirus-positive cells on liver biopsy due to disseminated adenovirus C infection [11]. Additionally, adenovirus hepatitis has presented as tumoral lesions on computed tomography (CT) scans, concerning for metastatic disease in a 59-year-old man with T-cell prolymphocytic leukemia [12]. Thus, although coagulative hepatocyte necrosis is the most common pathological manifestation of adenovirus hepatitis, multiple imaging and pathological features have been described.

Among the known species, adenovirus C has been most frequently isolated from immunocompromised patients, likely due to its ability to persist latently in lymphoid cells and reactivate under immunological stress [13]. In a case series of adenovirus infection among pediatric liver transplant recipients, the most common serotypes were 1, 2, and 5, all of which are members of the C species. The median timeline from transplantation to the collection of a culture-positive specimen was 25.5 days, with all symptomatic cases occurring within three months after transplant [6], illustrating that this is predominantly an early post-transplant complication. Although adenovirus is a recognized cause of disease in the post-transplant period, guidelines from the American Society of Transplantation Infectious Diseases Community of Practice recommend against routine screening due to the common occurrence of asymptomatic DNAemia [2]. Furthermore, while most organ donors are screened for human immunodeficiency virus, EBV, CMV, hepatitis B virus, and hepatitis C virus, and a minority of donors are screened for West Nile virus, human herpesvirus 8, Zika virus, and human T lymphotropic virus [14], adenovirus is not typically included in pretransplant screening.

Given the propensity for adenovirus C to persist latently and the fact that the earliest viral detection occurred in the transplanted liver, we strongly considered the possibility of a reactivation of a donor-derived infection in this case and recommended reporting to the United Network for Organ Sharing as such. Several lines of evidence support this possibility. First, such transmission has been previously documented to occur through solid organ transplantation [4,5], and in a study of pediatric liver transplant recipients, donor seropositivity with recipient seronegativity was documented in five of ten cases of adenovirus hepatitis [15]. Second, initial manifestations of disease occurred in the liver by hospital day six, while respiratory viral testing was negative on hospital day two. Though subsequent respiratory testing was positive, the negative initial test raises the possibility that the respiratory tract may not have been the primary site of infection [3]. Earlier biopsies of the donor liver and pretransplant donor and recipient serological testing may have provided more conclusive evidence of a nonrespiratory primary source, but no additional specimens were available for testing. To our knowledge, dissemination due to reactivation in the graft liver has not been definitively documented in cases of isolated liver transplantation. However, in a case series of culture-proven adenovirus enteritis in pediatric small bowel or composite liver-small bowel transplant recipients, four cases with adenovirus detection in both the graft and the respiratory tract were identified [16]. In all cases, detection in the graft preceded detection in the respiratory tract, suggesting that the graft was likely the initial site of infection followed by dissemination to other host tissues and highlighting the need for testing of multiple specimen types. Other potential modes of infection include reactivation of a latent recipient infection or a newly diagnosed acquired acute infection. Reactivation of a latent infection either from the recipient and/or donor organ seems most likely given the temporal correlation with periods of high immunosuppression in the early post-transplant period, which is a known risk factor for adenovirus reactivation [2]. High-dose steroids used to treat rejection in this case likely exacerbated disease, leading to recurrent fevers and high-grade DNAemia. Though an acute infection is possible, this case occurred in March 2021 during a time when the circulation of many common respiratory pathogens, including adenovirus, was substantially reduced due to nonpharmaceutical interventions enacted to control SARS-CoV-2 circulation [17], making acute infection less likely.

This case also highlights the utility of a nontargeted metagenomics-based test to facilitate the diagnosis of unusual presentations of common infections. Clinical metagenomic sequencing is currently available for testing of plasma, cerebrospinal fluid, and respiratory secretions for many human pathogens [18], and our patient’s infection was first detected by plasma cell-free DNA next-generation sequencing. While we regret that dedicated adenovirus PCR was not obtained earlier in the course for evaluation of elevated transaminases, the presentation with focal mass-like lesions with granulomatous infection suggested a need to consider a broader differential of pathogens, including fungal and mycobacterial organisms, as other possible causes. In our patient, Torque teno virus type 1 DNA was also found above threshold levels, but this was thought to be a likely incidental finding indicative of his degree of immunosuppression [19]. The lack of other pathogens identified on microbiological testing and the improvement of fevers following initiation of cidofovir supported the argument that adenovirus was pathogenic in this case, despite the absence of characteristic inclusions.

## Conclusions

In summary, this case provides an example of an unusual presentation (multifocal liver masses) of a common viral infection and demonstrates how disease manifestations may differ and how diagnostic evaluation and the treatment plan need to be adapted in highly immunocompromised hosts. This case also illustrates the potential for adenovirus to cause disease via reactivation of latent viral infection in solid organ transplant recipients, either from past infection of the recipient or from the transplanted organ, and raised awareness of this as a potential donor-transmitted infection among our transplant team. Finally, this case highlights a potential application of metagenomic next-generation sequencing of cell-free microbial DNA to expand the differential diagnosis in patients at risk for unusual or opportunistic infections and the use of complementary methods including histopathology to confirm the diagnosis.

## References

[1] Kwong AJ, Ebel NH, Kim WR (2023). OPTN/SRTR 2021 annual data report: liver. Am J Transplant.

[2] Florescu DF, Schaenman JM (2019). Adenovirus in solid organ transplant recipients: guidelines from the American Society of Transplantation infectious diseases community of practice. Clin Transplant.

[3] Al-Heeti OM, Cathro HP, Ison MG (2022). Adenovirus infection and transplantation. Transplantation.

[4] Pettengill MA, Babu TM, Prasad P (2019). Probable donor-derived human adenovirus type 34 infection in 2 kidney transplant recipients from the same donor. Open Forum Infect Dis.

[5] Kozlowski T, Nickeleit V, Andreoni K (2011). Donor-transmitted adenovirus infection causing kidney allograft nephritis and graft loss. Transpl Infect Dis.

[6] Michaels MG, Green M, Wald ER, Starzl TE (1992). Adenovirus infection in pediatric liver transplant recipients. J Infect Dis.

[7] McGrath D, Falagas ME, Freeman R, Rohrer R, Fairchild R, Colbach C, Snydman DR (1998). Adenovirus infection in adult orthotopic liver transplant recipients: incidence and clinical significance. J Infect Dis.

[8] Gutierrez Sanchez LH, Shiau H, Baker JM (2022). A case series of children with acute hepatitis and human adenovirus infection. N Engl J Med.

[9] Veer M, Abdulmassih R, Como J, Min Z, Bhanot N (2017). Adenoviral nephritis in a renal transplant recipient: case report and literature review. Transpl Infect Dis.

[10] Kelgeri C, Couper M, Gupte GL (2022). Clinical spectrum of children with acute hepatitis of unknown cause. N Engl J Med.

[11] Lerner AM, Bennett JE, Pittaluga S (2019). Protracted course of disseminated adenovirus disease with necrotizing granulomas in the liver. Diagn Microbiol Infect Dis.

[12] Putra J, Suriawinata AA (2014). Adenovirus hepatitis presenting as tumoral lesions in an immunocompromised patient. Ann Hepatol.

[13] Garnett CT, Erdman D, Xu W, Gooding LR (2002). Prevalence and quantitation of species C adenovirus DNA in human mucosal lymphocytes. J Virol.

[14] Nam H, Nilles KM, Levitsky J, Ison MG (2018). Donor-derived viral infections in liver transplantation. Transplantation.

[15] Koneru B, Atchison R, Jaffe R, Cassavilla A, Thiel DH, Starzl TE (1990). Serological studies of adenoviral hepatitis following pediatric liver transplantation. Transplant Proc.

[16] Pinchoff RJ, Kaufman SS, Magid MS (2003). Adenovirus infection in pediatric small bowel transplantation recipients. Transplantation.

[17] Olsen SJ, Winn AK, Budd AP (2021). Changes in influenza and other respiratory virus activity during the COVID-19 pandemic-United States, 2020-2021. Am J Transplant.

[18] Chiu CY, Miller SA (2019). Clinical metagenomics. Nat Rev Genet.

[19] Béland K, Dore-Nguyen M, Gagné MJ, Patey N, Brassard J, Alvarez F, Halac U (2014). Torque Teno virus in children who underwent orthotopic liver transplantation: new insights about a common pathogen. J Infect Dis.

